# Distribution and heritability of diurnal preference (chronotype) in a rural Brazilian family-based cohort, the Baependi study

**DOI:** 10.1038/srep09214

**Published:** 2015-03-18

**Authors:** Malcolm von Schantz, Tamara P. Taporoski, Andréa R. V. R. Horimoto, Nubia E. Duarte, Homero Vallada, José E. Krieger, Mario Pedrazzoli, André B. Negrão, Alexandre C. Pereira

**Affiliations:** 1 Faculty of Health and Medical Sciences, University of Surrey, Guildford, Surrey, UK; 2 Laboratory of Genetics and Molecular Cardiology, Heart Institute (Incor), University of São Paulo Medical School, São Paulo, SP, Brazil; 3 Department and Institute of Psychiatry (LIM 23), University of São Paulo Medical School, São Paulo, SP, Brazil; 4 School of Arts, Science, and Humanities, University of São Paulo, São Paulo, SP, Brazil

## Abstract

Diurnal preference (chronotype) is a useful instrument for studying circadian biology in humans. It harbours trait-like dimensions relating to circadian period and sleep homeostasis, but also has ontogenetic components (morningness increases with age). We used the Morningness-Eveningness questionnaire (MEQ) in the Baependi study, a family-based cohort study based in a small town in Minas Gerais, Brazil. The population is highly admixed and has a cohesive and conservative lifestyle. 825 individuals (497 female) aged 18–89 years (average ± SD = 46.4 ± 16.3) and belonging to 112 different families participated in this study. The average MEQ score was 63.5 ± 11.2 with a significant (P < 0.0001) linear increase with age. Morningness was significantly (P < 0.0001) higher in the rural (70.2 ± 9.8) than in the municipal zone (62.6 ± 11.1), and was also significantly (P = 0.025) higher in male (64.6 ± 10.9) than in female (62.8 ± 11.2) participants. Thus, in spite of universal access to electricity, the Baependi population was strongly shifted towards morningness, particularly in the rural zone. Heritability of MEQ score was 0.48 when adjusted for sex and age, or 0.38 when adjusted for sex, age, and residential zone. The reported MEQ score heritability is more akin to those of previous twin studies than previous family studies.

Circadian period is viewed as a stable and reproducible quantitative trait in humans^1^. It has been found to have a high degree of heritability in all vertebrates where it has been studied^2^,^3^,^4^, and super-short periods have been shown to co-segregate with single-gene mutations both in humans^5^ and in model animals^6^. Whilst circadian period may be determined experimentally in humans, most feasibly through the forced desynchrony protocol^7^, it is costly, intrusive, and time-consuming, and thus not a feasible option for large-scale human phenotyping. A more practical proxy for such studies is diurnal preference or chronotype, a self-reported questionnaire-based instrument that yields a numerical score on a scale ranging between extreme morning preference and extreme evening preference. It has been shown to correlate significantly not only with circadian phase, but also with circadian period^8^. However, the trait-like dimension of diurnal preference/chronotype is more complex, as in some individuals, morning versus evening preference results from differences in the build-up or dissipation of homeostatic sleep pressure rather than in circadian parameters^9^,^10^,^11^.

In addition to its endophenotypic qualities, chronotype is also influenced by state-like variables, some of them of considerable interest to our understanding of the interaction between the circadian oscillator and the sleep homeostat and our external environment^12^. There is a firmly established relationship between chronotype and age, with a peak in eveningness in late adolescence^13^ followed by a gradual increase in morningness^14^ reflecting an altered relationship between the circadian system and sleep-wake timing^8^.

The Morningness-Eveningness Questionnaire (MEQ) designed by Horne and Östberg^15^ is by far the most frequently employed chronotype instrument^16^,^17^. In the current study, the MEQ was administered to participants in a family-based cohort study based in Baependi, a small rural town (population: 18,307^18^) in the state of Minas Gerais in Brazil (21.95° S, 44.88° W). Baependi is a traditional community, with a cohesive culture and very limited migration. At the 2010 census, no individual living in the town was born in another country, and 99.0% of the population was classified as being born in the Southeast region of Brazil. Medium monthly income was R$818 for men and R$604 for women; median income was R$510 for both sexes. 28.1% of inhabitants aged 60 or above and 7.7% of those aged between 24 and 59 years old were illiterate. 93.9% of households had television, and 19.1% had internet access. Although 74.7% of the population described themselves as “white”, the population is profoundly admixed, almost entirely along the European-African axis. The demographic characteristics of the population and the traditional aspects of their lifestyle that have been lost in more developed areas made Baependi an ideal location for a family-based cohort study, which commenced in 2005 with a primary focus on cardiovascular health and disease^19^. The study has gradually grown to encompass a wider range of phenotypes related to health and well-being, one of which is presented here.

## Results

**Figure 1:**
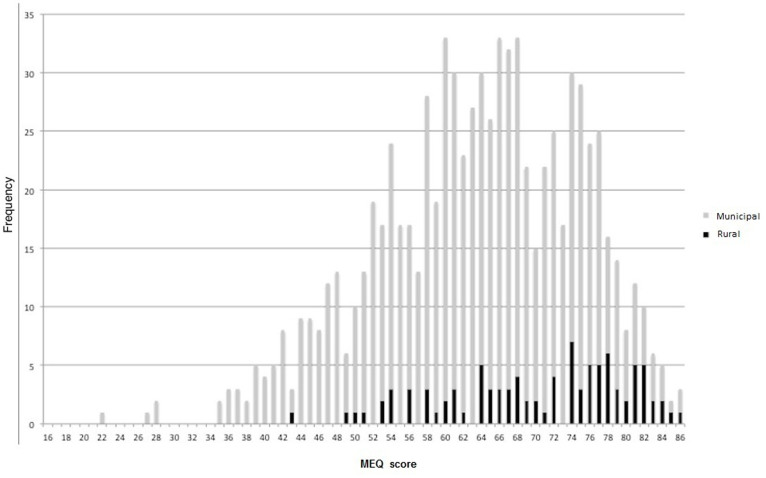
Histogram showing the distribution of MEQ score in the Baependi population. The black section of each bar for each score represents the frequency in the rural zone, and the remainder in the municipal zone. Low scores represent evening types and high scores morning types.

Table 1 shows the demographic characteristics of the population and the average total MEQ score, as well as the average stated preferred wake time and bedtime for the municipal and rural populations. The distribution of MEQ score in the urban and rural populations of Baependi is shown in Figure 1. The average score in the total population was 63.5 ± 11.2. Morningness was significantly higher in the rural (70.2 ± 9.8) than in the municipal zone (62.6 ± 11.1) (unpaired t-test, P < 0.0001). The effect-size or Cohen's d value was 0.73 (95% CI 0.54, 0.97) revealing a "medium" value of d, which suggests that the difference between these two groups is moderate. Morningness was significantly higher (P = 0.025) in male (64.6 ± 10.9) than in female (62.8 ± 11.2) participants. In this case, Cohen's d value was 0.16 (95% CI 0.024, 0.30), revealing that the difference between the two groups was very small.

**Figure 2:**
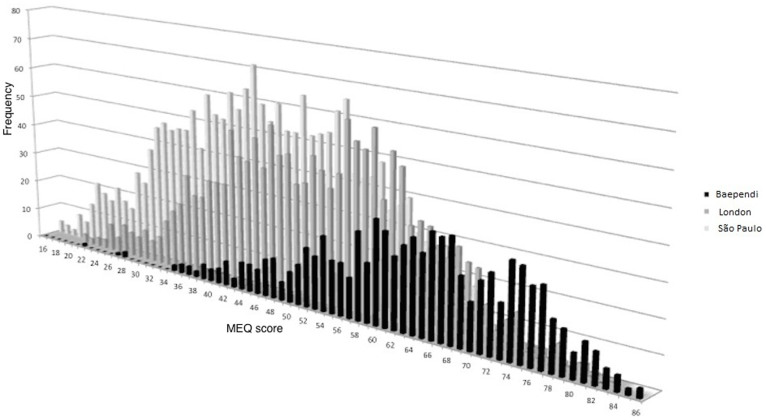
Histogram showing the distribution of MEQ score in the pooled Baependi population (black bars) contrasted with the distribution in the pooled data from two studies performed at the London Science Museum (dark grey bars) and one study performed within residents of the city of São Paulo (light grey bars).

The general shift towards morningness in the Baependi population as compared to urban ones is shown in Figure 2, where the combined data from all Baependi volunteers are plotted against the combined data from two studies performed in London^14^,^20^ as well as against data from residents of the city of São Paulo, collected through a web-based version of the validated Portuguese translation of the MEQ questionnaire.

**Figure 3:**
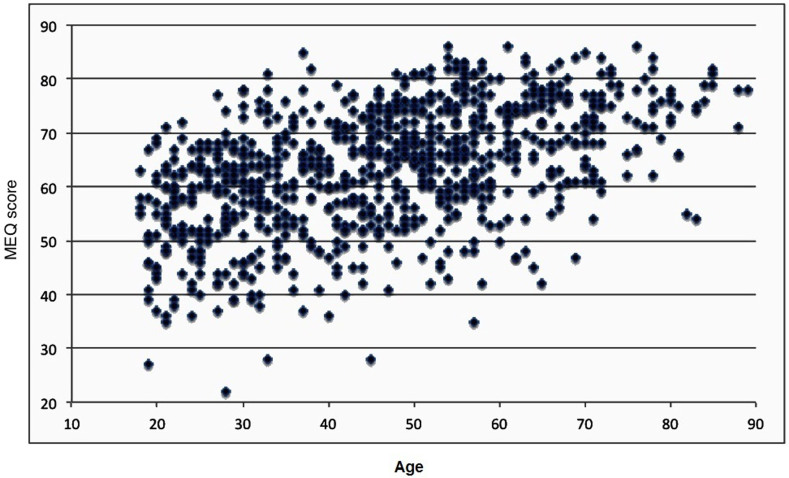
Distribution of MEQ score in the pooled Baependi population as a function of age.

**Figure 4:**
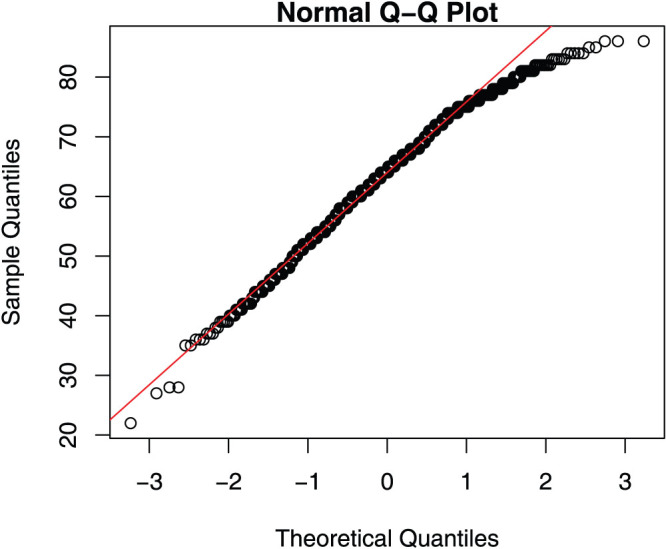
Normality plot of MEQ score distribution in the Baependi population. The high degree of normality across the medium and lower range of the scale is distorted at the highest range, representing extreme morningness.

Figure 3 shows MEQ score in the combined population as a function of age. As seen in this figure, the general morningness in the Baependi population was so high that, by age 50, more than 50% of them would be classified as extreme morning type based on the typology originally published with the scale (scores 70 to 86), and only one single subject beyond this age would be classified as a (moderate) evening type. The deviation from normality distribution of MEQ is illustrated in the normality plot (Figure 4), which clearly shows a deviation from the otherwise close fit to the regression line at higher MEQ scores.

The estimates of heritability (h^2^ ± standard error) are shown in Table 2. The heritability estimate for MEQ was 0.21 in the unadjusted or reduced mixed model. However, the inclusion of age and gender as covariates raised this value to 0.48. The added inclusion of age × age and age × gender did not change this value. However, when age, gender, and municipal versus rural residence were all added as covariates, heritability was calculated as 0.38.

## Discussion

Comparison between rural and municipal residents in Baependi (Figure 1) and between the Baependi population as a whole and previously collected data from a more urban population sampled at the London Science museum and another one sampled in the city of São Paulo (Figure 2) illustrate how strongly the distribution of diurnal preference/chronotype is affected by factors associated with residence, with urbanization shifting the distribution from greater morningness towards greater eveningness. The observable difference is likely slightly exaggerated by the fact that the other two populations were younger than the Baependi one (average age 36.4 in London and 31.8 in São Paulo); however, this age difference is entirely insufficient to account for the major part of the variance (the London study indicates that the relationship between age and MEQ score corresponds to an average increase of 1 unit approximately every 3.8 years^14^). The difference in chronotype between rural and metropolitan populations is well known and have been described in human population samples living both in Brazil^21^,^22^,^23^ and elsewhere^24^,^25^, and even in songbirds^26^. The most obvious and likely strongest determinant of this difference is differential exposure to natural light during the day (particularly during the advancing window of the phase response curve) and artificial light (from indoor or outdoor sources) during the night (particularly during the delaying window of the phase response curve). Indeed, the magnitude of the difference is quite similar to the shift reported in subjects studied first under an artificial light cycle and subsequently in the absence of all but natural light^27^. Other factors that could potentially contribute to the observed difference include a greater prevalence of hard physical labour in rural than in metropolitan areas, and the possibility that a rural individual with limited formal education (or even none at all), and limited experience of the outside world, may be unable to think abstractly about a life of leisure when answering questions such as preferred rise- and bedtime if they were completely free to plan their day. It also cannot be ruled out that the use of a scribe for recording the responses from a significant proportion of the participants has in some way affected the distribution of the answers. However, the answers to these questions would obviously relate to the subject's circadian phase. The fact that the metropolitan São Paulo population (located 1.60° S and 1.76° W of Baependi) shown in Figure 2 is much more similar to the London population does not suggest that the morningness of the Baependi population is based either on photoperiod or on national culture.

Previous reports on gender difference in MEQ score have been contradictory. The finding of greater morningness in men than in women in this population corroborates a recent report that the ontogeny of MEQ score in men and women follows different trajectories, and that an initial greater morningness in women is reversed around age 35^28^. In contrast to a number of previous studies showing greater morningess in women, which were based on samples of university students^29^,^30^, the average age in the Baependi study was 46.4 years.

As is already well known^8^, we found morningness to increase with age (Figure 3). However, our observations clearly demonstrate a specific limitation with the MEQ, namely that the scale cannot accommodate the full degree of morningness present in this sample. The suggestion in Figure 3 that the clustered distribution around an assumed linear regression line is compressed at the higher end of the MEQ scale (greater morningness), which does not extend beyond a score of 86, is confirmed by the plot in the Figure 1 (x-axis) and also by the normality plot (Figure 4) which shows a deviation from the normal distribution at higher scores of MEQ.

It is already well known that the typology proposed with the publication of the scale^15^ fits the distribution in a young metropolitan population, but is not appropriate at higher ages^31^. Our data suggest that some of the options offered for some of the answers do not accommodate the actual preference of an individual whose phase is more attuned to solar time. On more than one occasion, our participants tried to modify these answers, for example the answer to Question 1 ("Approximately what time would you get up if you were entirely free to plan your day?"), where they indicated that the earliest option offered (0500) was later than their actual preference.

Based on data reported here and elsewhere, although the MEQ score is modulated by age and location/lifestyle, reflecting a state of entrained phase, it retains a substantial trait-like endophenotypic dimension. It is interesting to note that the heritability estimates obtained here are much more similar to those previously reported for twin studies, which has been estimated at 52% for MEQ score^32^ and 44–50% using other scales^33^,^34^,^35^ than the estimates of 23%^36^ and 21%^24^ for family-based studies (only the latter using the MEQ as an instrument). Whilst these family studies were also performed in close-knit (and inbred) communities showing extreme morningness, both communities are based on adherence to anabaptist faith principles where the use of electrically powered devices including television is limited. It is possible, therefore, that in these studies, intrinsic diurnal preference was being masked, in part, by religious adherence.

A number of associations between candidate gene polymorphisms and MEQ have already been reported^37^,^38^,^39^,^40^,^41^,^42^,^43^. Our results suggest that genome-wide association studies (GWAS) for diurnal preference/chronotype are feasible in principle, but also highlight an important limitation in terms of the striking context-based variability of the MEQ score. As shown in Figure 2, a score indicative of evening type in Baependi represents an intermediate type in London or in São Paulo, just as a morning type score in these large cities would represent an intermediate in Baependi. And even though the MEQ distribution in the London and the São Paulo samples are more similar to each other than to that of Baependi, the pooling of samples from different populations that is currently adding considerable power to GWAS studies^44^ may not be feasible for diurnal preference/chronotype without matching of populations or transformation of scores^45^. However, this study shows that, within a well-defined cohort such as the Baependi one, there is a significant heritable dimension to the MEQ score that would make the GWAS approach feasible.

## Methods

The study protocol conformed to the tenets of the Declaration of Helsinki, and was approved by the ethics committee of the Hospital das Clínicas, University of São Paulo, Brazil. Each subject provided informed written consent before participation.

The recruitment methodology for the Baependi study has been described previously^19^. Briefly, probands were selected at random across 11 out of the 12 census districts in Baependi. After enrolment, the proband's first-degree (parents, siblings, and offspring), second-degree (half-siblings, grandparents/grandchildren, uncles/aunts, nephews/nieces, and double cousins), and third-degree (first cousins, great uncles/aunts, and great nephews/nieces) relatives, and his/her respective spouse's relatives resident both within Baependi (municipal and rural area) and surrounding towns were invited to participate. Only individuals age 18 and older were eligible to participate in the study. The study is conducted from a clinic/office in an easily accessible sector of the town, where the questionnaires were completed.

Prior to participating in this part of the study, participants had undergone a general health screening as well as providing multiple biochemical and physiological data points pertaining to cardiovascular health. The Portuguese version of the Morningness-Eveningness Questionnaire^46^ was administered as part of a booklet of questionnaires also pertaining to cognitive performance, psychiatric disorders, compulsive eating, and spirituality. Data were collected between April 5, 2013 and April 11, 2014. The questionnaire was completed in hard copy format, either by the participant or by a trained scribe. A small number of omissions of answers to single questions were rectified by telephoning the participant and repeating the question. Answers to individual questions were entered in a database together with the subject's age at the time, gender, and residence within the municipal or the rural zone of the town. Where residential zone was not explicitly noted as part of the address, the presence or absence of a street with a number was used to determine whether the participant was resident in the municipal or the rural zone.

The basic descriptive statistical including means and standard deviations, were estimated using the R program (http://www.R-project.org/). The variance component model, a well-known tool for heritability estimates in family studies, was used to calculate polygenic heritability estimates for MEQ score using the approach implemented in the kinship2 package (R package version 1.5.7, http://CRAN.R-project.org/package=kinship2) in the R software environment.

In the most narrow sense, the heritability of a trait represents the proportion of the phenotypic variance attributable to addictive genetic effects and is given by h^2^ = σ^2^_a_/σ^2^_p_, where σ^2^_a_ is the variance due to the addictive effects of genes, and σ^2^_p_ is the phenotypic variance. The overall phenotypic variance was estimated from the observed distribution of trait values in the sample, and was partitioned into genetic and environmental components using the observed covariance among family members, as Ω = 2Φ σ^2^_a_ + Iσ^2^_e_, where Ω is an nxn matrix of the n individuals in the data set, 2Φ is the structuring matrix of the coefficient of relationship, and I is an identity matrix that represents the structuring matrix for σ^2^_e_, the variance due to residual environmental factors. Estimates of the mean and variance components were obtained using maximum likelihood methods^47^,^48^,^49^. In a practical way, the heritability measures the level of correspondence between the phenotype and its genetic component. The function of heritability in genetic studies refers to its predictive role, expressing the confidence of the phenotypic value as a guide to justify more advanced genetic research^50^,^51^. Four models were fitted to the data: (1) Unadjusted, (2) adjusted by age and gender, (3) adjusted by age, gender, age^2^, and age and gender interaction, and (4) adjusted by age, gender and place of residence (municipal versus rural). The results are described in Table 2.

## Author Contributions

M.v.S., J.E.K., M.P., H.V., A.B.N. and A.C.P. designed the research, M.v.S., T.P.T., A.R.V.R.H., N.E.D. and A.B.N. performed it, M.v.S., A.R.V.R.H. and N.E.D. analysed the data, M.v.S., T.P.T., A.R.V.R.H., N.E.D., M.P., A.B.N. and A.C.P. wrote the main manuscript text. M.v.S. prepared Figures 1–3 and N.E.D. prepared Figure 4. All authors reviewed the manuscript.

## Figures and Tables

**Table 1:** Summary of demographic and experimental data from the sampled population

Population	Municipal	Rural	Total
Number	729	96	825
Average age	46.3 ± 16.4	47.7 ± 15.1	46.4 ± 16.3
Proportion of women	60.0%	62.5%	60.2%
Average MEQ score	62.6 ± 11.1	70.2 ± 9.8	63.5 ± 11.2
Average preferred wake time (question 1)	7.26 ± 1.42	6.46 ± 1.43	7.17 ± 1.45
Average preferred bed time (question 2)	22.29 ± 1.68	21.33 ± 1.13	22.18 ± 1.66

**Table 2:** Heritability of MEQ according to polygenic model adjusted for different covariates

Covariates	N	h^2^ ± SE	P-value
None	825	0.21 ± 0.07	1.9 e-4
Sex, age	822	0.48 ± 0.08	8.6 e-11
Sex, age, age^2^,sex * age	822	0.48 ± 0.08	5.9 e-11
Sex, age, residence	821	0.38 ± 0.09	3.0 e-6
